# Nitrite-Reductase and Peroxynitrite Isomerization Activities of *Methanosarcina acetivorans* Protoglobin

**DOI:** 10.1371/journal.pone.0095391

**Published:** 2014-05-14

**Authors:** Paolo Ascenzi, Loris Leboffe, Alessandra Pesce, Chiara Ciaccio, Diego Sbardella, Martino Bolognesi, Massimo Coletta

**Affiliations:** 1 Interdepartmental Laboratory of Electron Microscopy, University Roma Tre, Roma, Italy; 2 National Institute of Biostructures and Biosystems, Roma, Italy; 3 Department of Physics, University of Genova, Genova, Italy; 4 Department of Clinical Sciences and Translational Medicine, University of Roma “Tor Vergata”, Roma, Italy; 5 Interuniversity Consortium for the Research on the Chemistry of Metals in Biological Systems, Bari, Italy; 6 Department of Bioscience, University of Milano, Milano, Italy; Instituto de Tecnologia Quimica e Biologica, Portugal

## Abstract

Within the globin superfamily, protoglobins (Pgb) belong phylogenetically to the same cluster of two-domain globin-coupled sensors and single-domain sensor globins. Multiple functional roles have been postulated for *Methanosarcina acetivorans* Pgb (Ma-Pgb), since the detoxification of reactive nitrogen and oxygen species might co-exist with enzymatic activity(ies) to facilitate the conversion of CO to methane. Here, the nitrite-reductase and peroxynitrite isomerization activities of the CysE20Ser mutant of Ma-Pgb (Ma-Pgb*) are reported and analyzed in parallel with those of related heme-proteins. Kinetics of nitrite-reductase activity of ferrous Ma-Pgb* (Ma-Pgb*-Fe(II)) is biphasic and values of the second-order rate constant for the reduction of NO_2_^–^ to NO and the concomitant formation of nitrosylated Ma-Pgb*-Fe(II) (Ma-Pgb*-Fe(II)-NO) are *k*_app1_ = 9.6±0.2 M^–1^ s^–1^ and *k*_app2_ = 1.2±0.1 M^–1^ s^–1^ (at pH 7.4 and 20°C). The *k*_app1_ and *k*_app2_ values increase by about one order of magnitude for each pH unit decrease, between pH 8.3 and 6.2, indicating that the reaction requires one proton. On the other hand, kinetics of peroxynitrite isomerization catalyzed by ferric Ma-Pgb* (Ma-Pgb*-Fe(III)) is monophasic and values of the second order rate constant for peroxynitrite isomerization by Ma-Pgb*-Fe(III) and of the first order rate constant for the spontaneous conversion of peroxynitrite to nitrate are *h*_app_ = 3.8×10^4^ M^–1^ s^–1^ and *h*_0_ = 2.8×10^–1^ s^–1^ (at pH 7.4 and 20°C). The pH-dependence of *h*_on_ and *h*_0_ values reflects the acid-base equilibrium of peroxynitrite (p*K*_a_ = 6.7 and 6.9, respectively; at 20°C), indicating that HOONO is the species that reacts preferentially with the heme-Fe(III) atom. These results highlight the potential role of Pgbs in the biosynthesis and scavenging of reactive nitrogen and oxygen species.

## Introduction

Phylogenetic analysis revealed that members of the globin superfamily evolved from an ancestral monomeric flavo-hemoglobin and were arranged in three globin lineages and two structural classes. The first lineage includes flavo-hemoglobins and related single domain globins, the second lineage embraces truncated hemoglobins, and the third lineage encompasses two-domain globin-coupled sensors, single-domain sensor globins and related single-domain protoglobins (Pgb) [1], [2]. All members of the first and third lineage belong to the same structural class, showing a long amino acid sequence (>145 residues) and the classical 3-on-3 α-helical fold found in myoglobin, with the heme group surrounded by the A, B, and E α-helices on one side and the F, G, and H α-helices on the other [3], [4]. Conversely, all members of the second lineage, consisting of three subgroups, belong to the same structural class and show a short amino acid sequence (<135 residues), adopting the 2-on-2 α-helical sandwich fold, characterized by a very short or absent A-helix, a brief CE inter-helical region and most of the F-helix occurring as a loop, with only the B, E, G, and H α-helices surrounding the heme [5].

To date, nine Pgbs have been identified in Archaea and Bacteria [1], [2], [6]–[9], however only two Pgbs from the obligate aerobic hyperthermophile *Aeropyrum pernix* and from the strictly anaerobic methanogen *Methanosarcina acetivorans* (*M. acetivorans* MaPgb) have been characterized [10].

**Figure 1:**
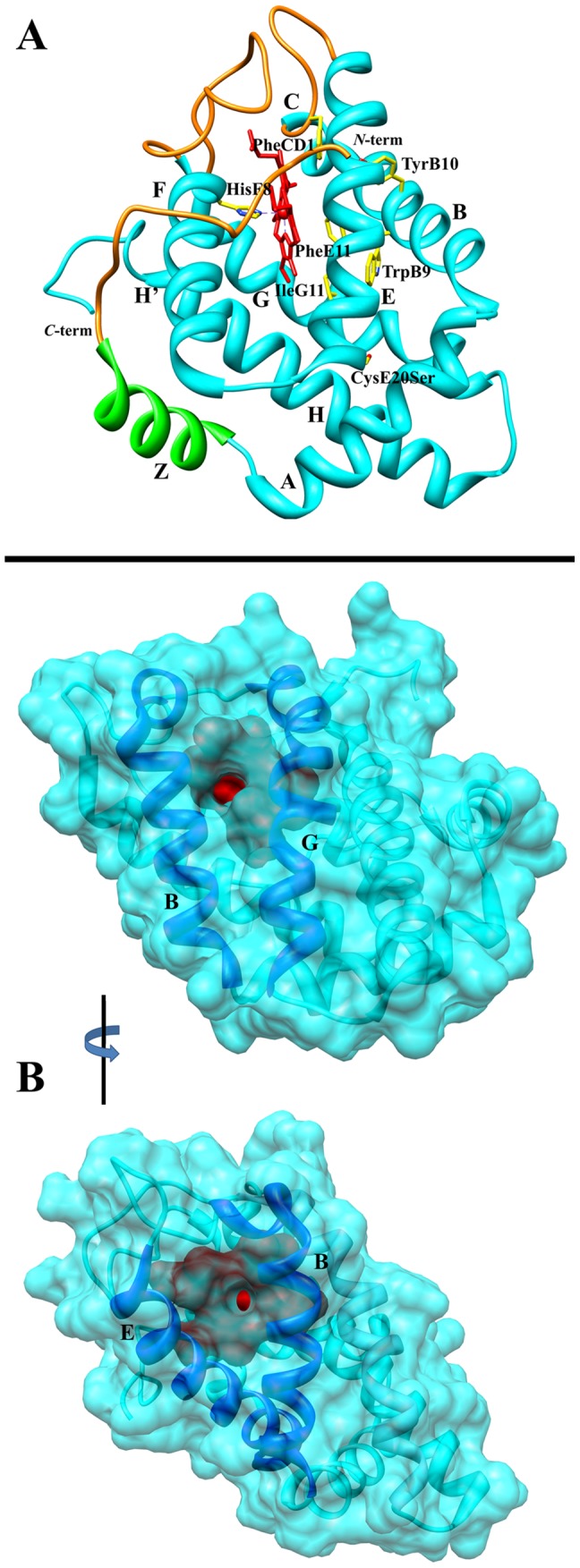
Ma-Pgb* fold. (Panel A) The secondary structure elements are labeled A–H’. The 20 *N*-terminal residues and the extended CE and FG loops that seal the heme pocket and prevent the access of small ligands to the heme distal cavity are in orange. The pre-A Z-helix is in green. The heme (red) is displayed edge on. The proximal HisF8 residue is shown on the left hand side of the heme. The picture includes the mutated SerE20 residue, located at the *C*-terminus of the E-helix. The HisF8 and SerE20 side chains and residues building up the heme distal pocket are drawn as skeletal models (C atoms yellow, N atoms blue, and O atoms red) and labeled. (Panel B) Mono views of “tunnel 1” (top) and “tunnel 2” (bottom) access sites in Ma-Pgb*. Helices flanking the tunnel entries are labelled. The heme group (seen through the tunnel apertures) is shown in red. The protein is correctly oriented in both images, to bring each tunnel in the direction of sight. The images are rotated by 90°. The pictures have been drawn by UCSF - Chimera [55]. For details, see ref. [11].

*Methanosarcina acetivorans* protoglobin (Ma-Pgb), generally taken as the molecular model of Pgbs, shows a homodimeric quaternary structure mostly based on the inter-molecular four-helix bundle built by the G and H α-helices of each protomer. The three-dimensional structure of the Ma-Pgb* monomer (a site-directed mutant of Ma-Pgb displaying the CysE20Ser mutation to prevent the formation of intermolecular disulphide bonds) shows that the 195 amino acid chain can be considered an expanded version of the classical 3-on-3 α-helical fold globin fold, being characterized by the presence of a 20-residue *N*-terminal extension and a pre-A α-helix Named Z-helix). Moreover, the heme of Ma-Pgb* is markedly distorted and fully buried in the protein matrix, due to extended CE and FG loops and the 20-residue *N*-terminal extension (Fig. 1, panel A). Therefore, the access of ligands to the heme distal pocket is granted by two protein matrix tunnels, which are located at the B/G (tunnel 1) and B/E (tunnel 2) α-helix interfaces (Fig. 1, panel B) [11]–[16].

Multiple functional roles have been postulated for Ma-Pgb, since the detoxification of reactive nitrogen and oxygen species, which appears pivotal in the physiology of the strictly anaerobe *Methanosarcina acetivorans*, might co-exist with enzymatic activities facilitating the conversion of acetate, methanol, CO_2_, and CO to methane [17]. As expected for a strict anaerobe, the generation of reduced Ma-Pgb* requires an O_2_-free environment as the heme-Fe(II) atom autoxidizes rapidly (t_1/2_ = 3.6 min) [7], [8]. On the other hand, ferric Ma-Pgb* (Ma-Pgb*-Fe(III)) undergoes reductive nitrosylation through the transient formation of the ferric nitrosylated species, which is converted to the ferrous form that in turn binds NO very rapidly [16].

Here, kinetics of the NO_2_^–^-mediated conversion of ferrous Ma-Pgb* (Ma-Pgb*-Fe(II)) to the ferrous nitrosylated derivative (Ma-Pgb*-Fe(II)-NO) and of the ferric Ma-Pgb*-Fe(III)-mediated peroxynitrite isomerization are reported and analyzed in parallel with those of related heme-proteins to highlight the potential role of Pgbs in the biosynthesis and scavenging of reactive nitrogen and oxygen species.

## Materials

Ma-Pgb*-Fe(III) was expressed in *Escherichia coli* cells Bl21(DE3)pLysS, collected, and purified as previously reported [11], [15]. The analysis of the structure of Ma-Pgb*-Fe(II) and Ma-Pgb*-Fe(III) derivatives indicates that the SerE20 residue is not involved in any specific interaction critical for structural stability, but is partly exposed to the solvent (see Fig. 1). Moreover, SerE20 falls at the *C*-terminal end of the E-helix, in a location 18 Å away from the heme-ligand binding site, from which this residue is isolated by the protein matrix (see Fig. 1). Thus, the CysE20Ser mutation is justifiable to ease crystallization [14], [17], [18]. Furthermore, values the second-order rate constants for CO binding to ferrous wild type Ma-Pgb and Ma-Pgb*, obtained by rapid-mixing stopped-flow and laser photolysis techniques, are closely similar [11], [15]. The different values of the first-order rate constants for CO dissociation from ferrous carbonylated Ma-Pgb and Ma-Pgb*, obtained by different methods based on the conversion of the CO-bound protein to the NO-bound form (*i.e.*, by mixing the carbonylated protein with dithionite/nitrite or the NO donor MAHMA NONOate solution) [11], [15], may reflect different steps or mechanisms of CO escaping from the heme pocket to the solvent.

The Ma-Pgb*-Fe(III) concentration was determined spectrophotometrically using the extinction coefficient at 399 nm (*i.e.*, ε = 1.38×10^5^ M^−1^ cm^−1^), pH 7.0 (5.0×10^−2^ M 1,3-bis(tris(hydroxymethyl)methylamino)propane (bis-tris-propane) buffer) and 20°C (see [18] and present study). Ma-Pgb*-Fe(II) was obtained, under anaerobic conditions, by adding Na_2_S_2_O_4_ (final concentration, 1.0×10^–3^ M to 5.0×10^–3^ M). The Ma-Pgb*-Fe(II) concentration was determined spectrophotometrically using the pH-independent (5.0×10^−2^ M bis-tris-propane buffer) extinction coefficient at 432 nm (*i.e.*, ε = 1.25×10^5^ M^−1^ cm^−1^), 20°C [15].

Peroxynitrite was synthesized from KO_2_ and NO and from HNO_2_ and H_2_O_2_ and stored in small aliquots at −80°C [19], [20]. The peroxynitrite stock solution (2.0×10^−3^ M) was diluted immediately before use with degassed 5.0×10^−2^ M NaOH to reach the desired concentration [21]–[32]. The concentration of peroxynitrite was determined spectrophotometrically prior to each experiment by measuring the absorbance at 302 nm (ε_302 nm_ = 1.705×10^3^ M^−1^ cm^−1^) [19], [20]. The term “peroxynitrite” is used here to refer generically to both ONOO^−^ and its conjugate acid HOONO (see [22], [24], [28]).

CO was purchased from Linde AG (Höllriegelskreuth, Germany) or Rivoira (Milan, Italy). The CO stock solution was prepared by keeping anaerobically distilled water in a closed vessel under CO at *P* = 760.0 mm Hg (*T* = 20°C). The solubility of CO in water is 1.03×10^−3^ M, at *P* = 760.0 mm Hg and 20°C [33].

NO (from Aldrich Chemical Co., Milwaukee, WI, USA) was purified by flowing it through a NaOH column in order to remove acidic nitrogen oxides. Bis-tris propane, sodium azide (NaN_3_), sodium dithionite (Na_2_S_2_O_4_), sodium nitrite (NaNO_2_), and were obtained from Merck KGaA (Darmstadt, Germany).

All chemicals where of analytical grade and were used without further purification unless stated.

## Methods

### The NO_2_^–^-mediated Conversion of Ma-Pgb*-Fe(II) to Ma-Pgb*-Fe(II)-NO

Kinetics of NO_2_^–^-mediated conversion of Ma-Pgb*-Fe(II) to Ma-Pgb*-Fe(II)-NO, in the absence and presence of CO, was recorded with the SMF-20 rapid-mixing stopped-flow apparatus (Bio-Logic SAS, Claix, France). The light path of the observation cuvette was 10 mm, and the dead time was 1.4 ms. Kinetics was detected spectrophotometrically at single wavelengths between 380 nm and 460 nm, the wavelength interval was 2.5 nm.

Kinetic data were obtained in the absence and presence of CO (final concentration, 1.0×10^−4^ M), by rapid mixing the Ma-Pgb*-Fe(II) solution (final concentration, 3.8×10^−6^ M) with the NO_2_^–^ solution (final concentration, 1.0×10^−3^ M to 1.0×10^−2^ M), in the presence of sodium dithionite (final concentration, 2.0×10^–3^ M), between pH 6.2 and 8.3 (5.0×10^−2^ M bis-tris-propane buffer) and 20°C. No gaseous phase was present.

Although the sodium dithionite concentration lower than 1.0×10^–2^ M has been reported not to reduce significantly NO_2_^–^ to NO [34], the appropriate control was performed as follows. Five mL of the NO_2_^–^ solution (final concentration, 1.0×10^–2^ M) were reacted with 5.0 mL of the sodium dithionite solution (final concentration, 2.0×10^–3^ M), at pH 7.4 and 20°C for 10 min, under anaerobic conditions. Then, the concentration of NO_2_^–^ was determined spectrophotometrically at 543 nm by using the Griess reagent [35] and by reductive chemiluminescence [34]. The concentration of NO_2_^–^ determined was (9.8±0.9)×10^–3^ M. This indicates that in the presence of 1.0×10^–2^ M NO_2_^–^ and 2.0×10^–3^ M sodium dithionite, the reduction of NO_2_^–^ to NO does not take place. Moreover, if NO should have been produced, it would have reacted instantaneously with Ma-Pgb*-Fe(II) [16] impairing the slow NO_2_^–^-mediated conversion of Ma-Pgb*-Fe(II) to Ma-Pgb*-Fe(II)-NO.

Experiments in the presence of CO were carried out at pH 7.4 and 20°C by adding 1.0×10^−4^ M CO to the Ma-Pgb*-heme-Fe(II) and NO_2_^–^ solutions. This CO concentration allowed to obtain the fully saturated Ma-Pgb*-Fe(II)-CO complex, the value of the overall dissociation equilibrium constant being about 1×10^−8^ M [15].

The kinetics of NO_2_^–^-mediated conversion of Ma-Pgb*-Fe(II) to Ma-Pgb*-Fe(II)-NO in the absence and presence of CO was analyzed in the framework of the minimum reaction mechanism depicted by Scheme 1 [16], [34], [36]–[45]:
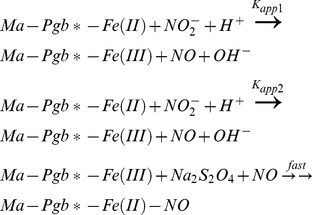



### Scheme 1

It is important to outline that the two processes described by *k*_app1_ and *k*_app2_ correspond to the same reaction for the two molecular species observed (see below). The process depicted by *fast* corresponds to the very fast nitrosylation of Ma-Pgb*-Fe(II) in the presence of sodium dithionite [16].

Values of the pseudo-first-order rate constant (*i.e.*, *k*_obs1_ and *k*_obs2_) and of the amplitude (*i.e.*, [Ma-Pgb*-Fe(II)]_i1_ and [Ma-Pgb*-Fe(II)]_i2_) of the fast and slow phases, respectively, for the NO_2_^–^-mediated conversion of Ma-Pgb*-Fe(II) to Ma-Pgb*-Fe(II)-NO were determined, in the absence and presence of CO (final concentration, 1.0×10^−4^ M), from data analysis, according to Eqs 1a and 1b, depending on the wavelength (*i.e.*, on the increase or the decrease of the spectral change; see Fig. S1) [36], [42], [43], [45], [46]:
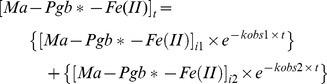
(1a)




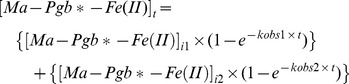
(1b)where [Ma-Pgb*-Fe(II)]_i1_+ [Ma-Pgb*-Fe(II)]_i2_ corresponds to the total amplitude (*i.e.*, [Ma-Pgb*-Fe(II)]_itot_ = α_1_×[Ma-Pgb*-Fe(II)]_i1_+ α_2_ × [Ma-Pgb*-Fe(II)]_i2_) with α_1+_ α_2_ = 1. Values of the second order rate constant for the NO_2_^–^-mediated conversion of Ma-Pgb*-Fe(II) to Ma-Pgb*-Fe(II)-NO (*i.e.*, *k*_app1_ and *k*_app2_) were obtained from the dependence of *k*_1_ and *k*_2_ on the NO_2_^–^ concentration (*i.e.*, [NO_2_^–^]), according to Eqs 2 and 3, respectively [36], [42], [43], [45], [46]:



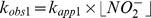
(2)

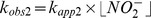
(3)


According to literature [36], [42], [43], [45], [46], the values of *k*_app1_ and *k*_app2_ refer to the interaction of NO_2_^–^ with Ma-Pgb-Fe*(II) under the general assumption that this process represents the rate-limiting step of the overall reaction reported in Scheme 1.

### The Ma-Pgb*-Fe(III)-mediated Peroxynitrite Isomerization

Kinetics of peroxynitrite isomerization in the absence and presence of Ma-Pgb*-Fe(III) and Ma-Pgb*-Fe(III)-azide was recorded with the SMF-20 rapid-mixing stopped-flow apparatus (Bio-Logic SAS, Claix, France). The light path of the observation cuvette was 10 mm, and the dead time was 1.4 ms. Kinetics was monitored spectrophotometrically at 302 nm, the characteristic absorbance maximum of peroxynitrite [19], [20]. Moreover, kinetics of peroxynitrite isomerization by Ma-Pgb*-Fe(III) was monitored spectrophotometrically at single wavelengths between 380 nm and 460 nm, the wavelength interval was 2.5 nm, to evaluate the transient formation of the Ma-Pgb*-Fe(III)-OONO species.

Kinetic data were obtained by rapid mixing either the Ma-Pgb*-Fe(III) solution (final concentration, 5.0×10^−6^ M to 5.0×10^−5^ M) or the Ma-Pgb*-Fe(III)-azide solutions (final concentrations, 5.0×10^−6^ M to 5.0×10^−5^ M) or the buffer solution (5.0×10^−2^ M bis-tris-propane buffer) with the peroxynitrite solution (final concentration, 2.5×10^−4^ M). Kinetics was obtained at 20°C and between pH 6.2 and 8.3; the pH was always measured at the end of the reaction. No gaseous phase was present.

Experiments in the presence of azide were carried out at pH 7.4 and 20°C by adding 1.0×10^−1^ M azide to the Ma-Pgb*-heme-Fe(III) and peroxynitrite solutions. This azide concentration allowed to obtain the fully saturated Ma-Pgb*-Fe(III)-azide complex, the value of the dissociation equilibrium constant being <1×10^−3^ M [18].

The kinetics of peroxynitrite isomerization in the absence and presence of Ma-Pgb*-Fe(III) and Ma-Pgb*-Fe(III)-azide was analyzed in the framework of the minimum reaction mechanisms depicted by Schemes 2 and 3, respectively [21]–[32]:
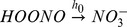



### Scheme 2



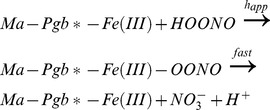



### Scheme 3

Values of the first-order rate constant for peroxynitrite isomerization in the presence Ma-Pgb*-Fe(III)-azide and 5.0×10^−2^ M bis-tris-propane buffer (*i.e.*, *h*_0_) and of the pseudo-first-order rate constant for peroxynitrite isomerization in the presence of Ma-Pgb*-Fe(III) (*i.e.*, *h*_obs_) have been determined from the analysis of the time-dependent absorbance decrease at 302 nm, according to Eq. 4 [21]–[32], [47], [48]:
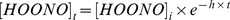
(4)where *h* is either *h*_0_ or *h*_obs_.

Values of the second-order rate constant for Ma-Pgb*-Fe(III)-mediated peroxynitrite isomerization (*i.e.*, *h*_on_) has been determined from the linear dependence of *h*_obs_ on the Ma-Pgb*-Fe(III) concentration, according to Eq. 5 [21]–[25], [27]–[32]:
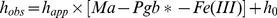
(5)


The pH dependence of *h*_0_ and *h*_app_ for peroxynitrite isomerization in the absence and presence of Ma-Pgb*-Fe(III), respectively, allowed us to obtain the values of p*K*_a_, *h*_(prot)_, and *h*_(unprot)_, according to Eq. 6 [22], [24], [28], [48]:
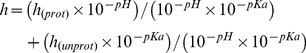
(6)where *h* is *h*_0_ or *h*_app_, *h*_(prot)_ represents the asymptotic value of *h* under conditions where pH <<p*K*_a_, and *h*_(unprot)_ represents the asymptotic value of *h* under conditions where pH >>p*K*_a_.

### Data Analysis

The results are given as mean values of at least four experiments plus or minus the corresponding standard deviation. All data were analyzed using the Matlab program (The Math Works Inc., Natick, MA, USA).

## Results

### The NO_2_^–^-mediated Conversion of Ma-Pgb*-Fe(II) to Ma-Pgb*-Fe(II)-NO

**Figure 2:**
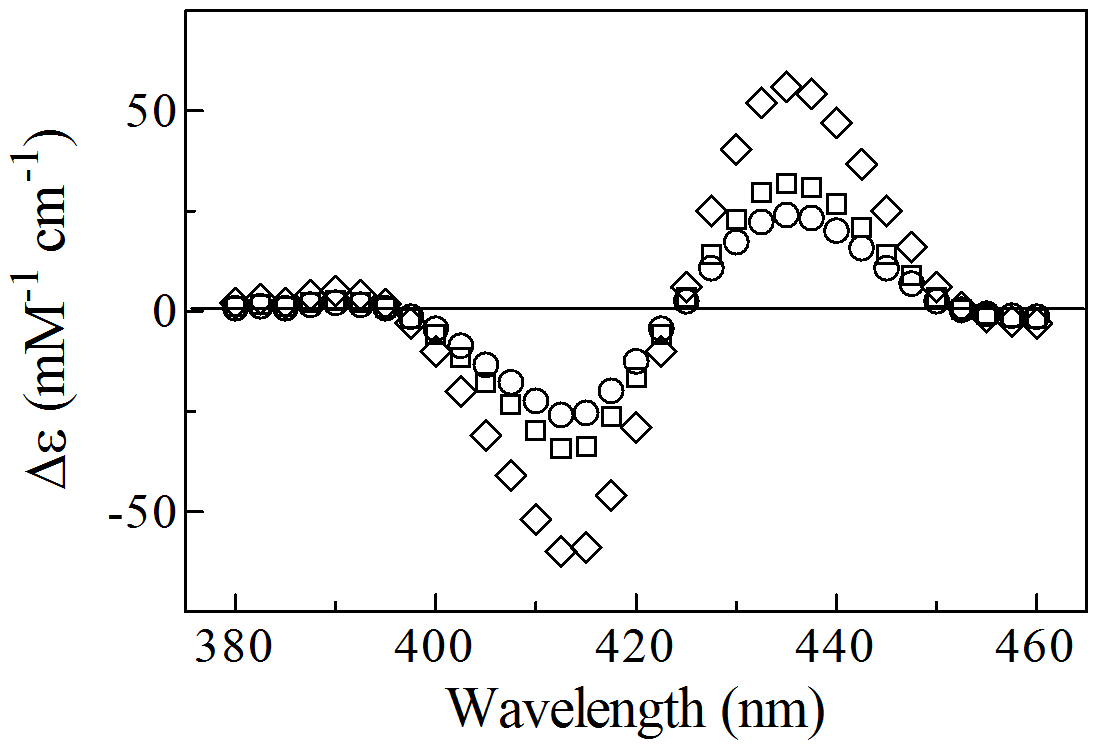
Difference absorbance spectra of Ma-Pgb*-Fe(II) *minus* Ma-Pgb*-Fe(II)-NO, at pH 7.4 and 20°C. The overall difference spectrum, the difference spectrum of the fast phase, and the difference spectrum of the slow phase are represented by diamonds, squares, and circles, respectively. For details, see text.

Mixing the Ma-Pgb*-Fe(II) and NO_2_^–^ solutions induces a shift of the optical absorption maximum of the Soret band from 432 nm (*i.e.*, Ma-Pgb*-Fe(II)) to 414 nm (*i.e.*, Ma-Pgb*-Fe(II)-NO) and a corresponding change of the extinction coefficient from ε_432 nm_ = 1.25×10^5^ M^–1^ cm^–1^ to ε_414 nm_ = 1.28×10^5^ M^–1^ cm^–1^. The absorbance spectrum of Ma-Pgb*-Fe(II)-NO obtained by mixing Ma-Pgb*-Fe(II) and NO_2_^–^ solutions corresponds to that obtained by adding gaseous NO to the Ma-Pgb*-Fe(II) and Ma-Pgb*-Fe(III) solutions [17]. Moreover, the absorbance spectra of the fast- and slow-reacting Ma-Pgb*-Fe(II) species are superimposable (Fig. 2).

**Figure 3:**
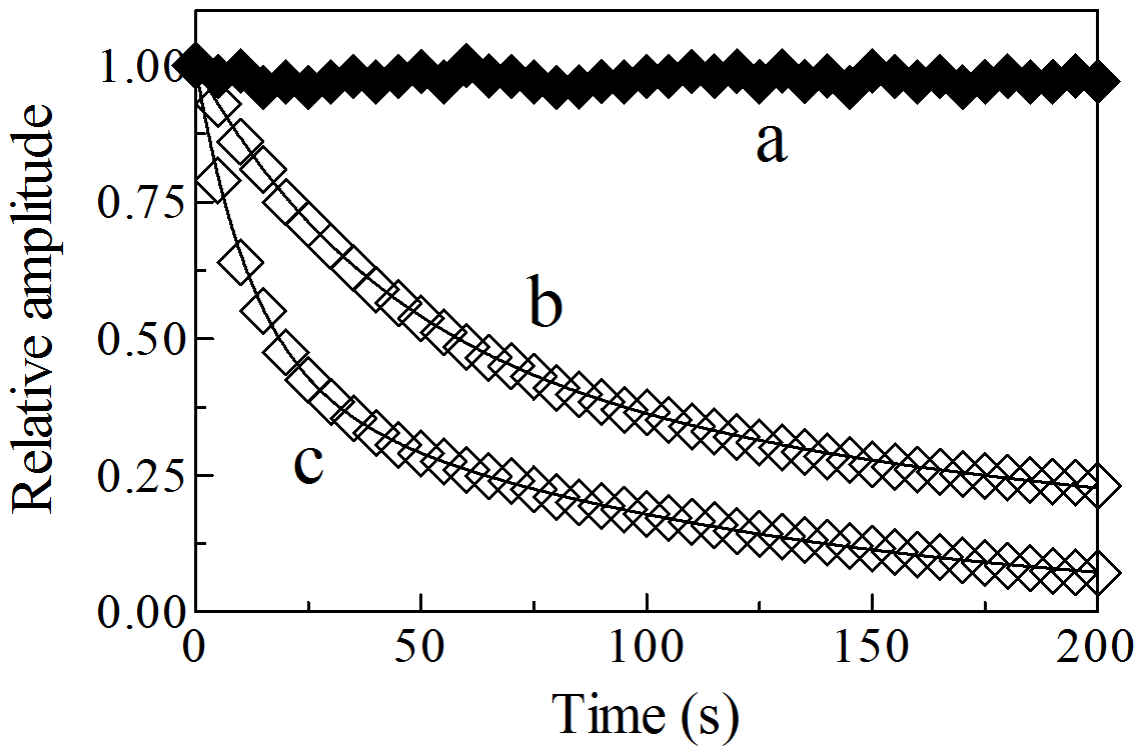
Normalized averaged time courses of the NO_2_^–^-mediated nitrosylation of Ma-Pgb*-Fe(II), in the absence and presence of CO, at pH 7.4 and 20°C, λ = 430 nm. The NO_2_^–^ concentration was 2.5×10^–3^ M (traces a and b) and 8.0×10^–3^ M (trace c). The CO concentration was 1.0×10^–4^ M (trace a). CO inhibits the NO_2_^–^-mediated nitrosylation of Ma-Pgb*-Fe(II) (trace a). The time course analysis according to Eqn. 1 allowed the determination of the following parameters: trace b, α_1_ = 0.58±0.05, *k*_obs1_ = (2.3±0.2) ×10^–2^ s^–1^, α_2_ = 0.42±0.04, and *k*_obs2_ = (3.2±0.3)×10^–3^ s^–1^; trace c, α_1_ = 0.56±0.05, *k*_obs1_ = (7.9±0.8)×10^–2^ s^–1^, α_2_ = 0.44±0.04, and *k*_obs2_ = (9.0±0.1)×10^–3^ s^–1^. For details, see text.

Under all experimental conditions, the time course of the NO_2_^–^-mediated conversion of Ma-Pgb*-Fe(II) to Ma-Pgb*-Fe(II)-NO corresponds to a biphasic process, the amplitude of the fast and of the slow phase corresponding to 57±5% and 43±5%, respectively, of the whole process, at all NO_2_^–^ concentrations (Fig. 3). Values of the pseudo-first-order rate constant for the NO_2_^–^-mediated conversion of Ma-Pgb*-Fe(II) to Ma-Pgb*-Fe(II)-NO (*i.e.*, *k*_1_ and *k*_2_; see Eq. 1) are wavelength-independent at fixed NO_2_^–^ concentration (data not shown). Of note (see [34] and present study), values of *k*_1_ and *k*_2_ are unaffected by the dithionite concentration between 1.0×10^–3^ M and 5.0×10^–3^ M (data not shown).

**Figure 4:**
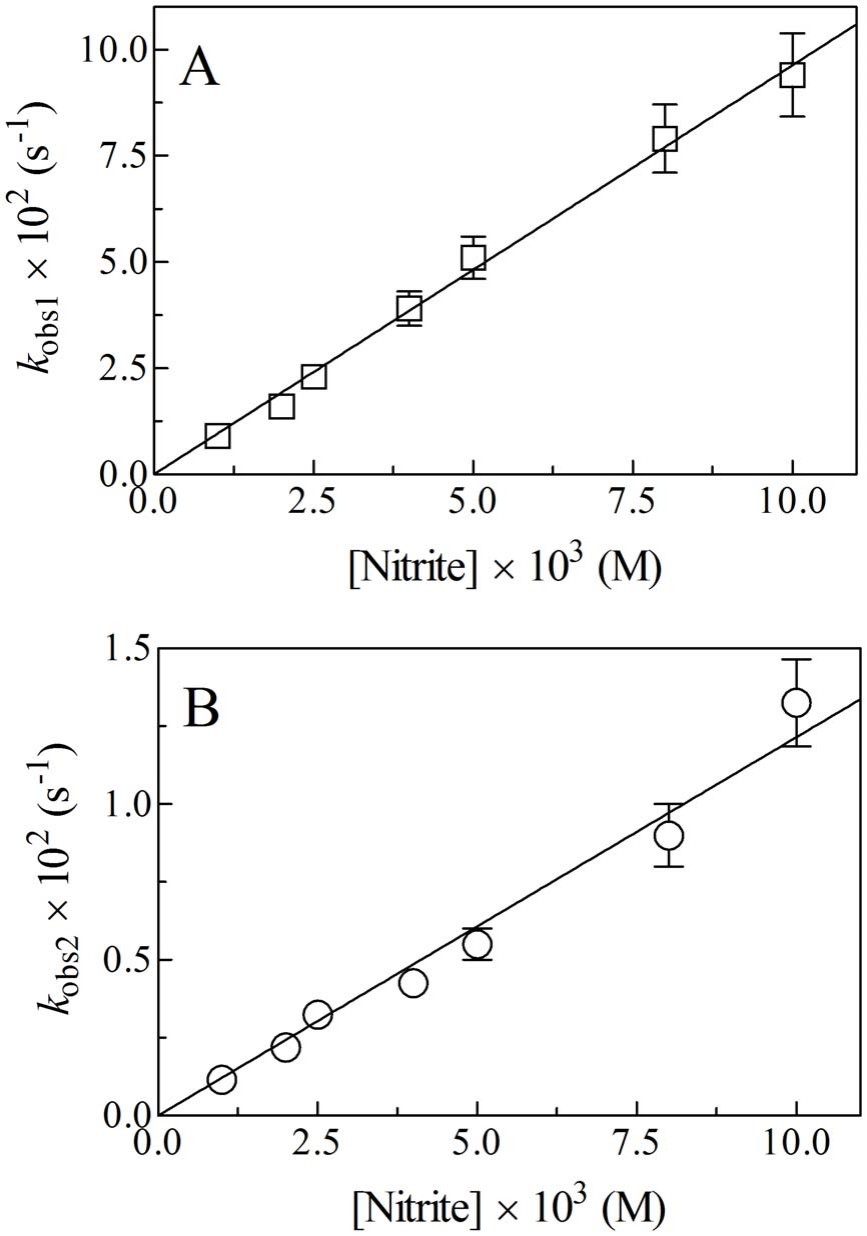
Dependence of *k*_obs1_ (panel A) and *k*_obs2_ (panel B) on the NO_2_^–^ concentration for the Ma-Pgb*-Fe(II) reductase activity, at pH 7.4 and 20°C. The analysis of data according to Eqs 2 and 3 allowed the determination of the following values of *k*_app1_ = 9.6±0.2 M^–1^ s^–1^ and *k*_app2_ = 1.2±0.1 M^–1^ s^–1^. Where not shown, the standard deviation is smaller than the symbol. For details, see text.

Values of *k*_app1_ and *k*_app2_ increase linearly with the NO_2_^–^ concentration (Fig. 4). The analysis of data reported in Fig. 4 according to Eqs 2 and 3 allowed the determination of values of the second-order rate constants for the NO_2_^–^-mediated conversion of Ma-Pgb*-Fe(II) to Ma-Pgb*-Fe(II)-NO (*i.e.*, *k*_app1_ = 9.6±0.2 M^–1^ s^–1^ and *k*_app2_ = 1.2±0.1 M^–1^ s^–1^; corresponding to the slope of the linear plots). The *y* intercept of the linear plots corresponds to zero, indicating that the NO_2_^–^-mediated conversion of Ma-Pgb*-Fe(II) to Ma-Pgb*-Fe(II)-NO can be considered as an irreversible process. Values of the rate constants for the NO_2_^–^-mediated conversion of Ma-Pgb*-Fe(II) to Ma-Pgb*-Fe(II)-NO are lower by several orders of magnitude than that of Ma-Pgb*-Fe(II) nitrosylation [16]. This indicates that the formation of the transient Ma-Pgb*-Fe(III) species (see Scheme 1), which is quickly converted to Ma-Pgb*-Fe(II) by reacting with dithionite, represents the rate-limiting step of the NO_2_^–^-mediated conversion of Ma-Pgb*-Fe(II) to Ma-Pgb*-Fe(II)-NO. NO_2_^–^ does not convert Ma-Pgb*-Fe(II)-CO to Ma-Pgb*-Fe(II)-NO (Fig. 3); the difference absorbance spectrum of Ma-Pgb*-Fe(II)-CO *minus* Ma-Pgb*-Fe(II)-NO is shown in Fig. S2. Therefore, the NO_2_^–^-mediated nitrosylation of Ma-Pgb*-Fe(II) reflects the reaction of NO_2_^–^ with the heme-Fe(II) atom, as reported for plant and cyanobacterial hemoglobins [42].

**Figure 5:**
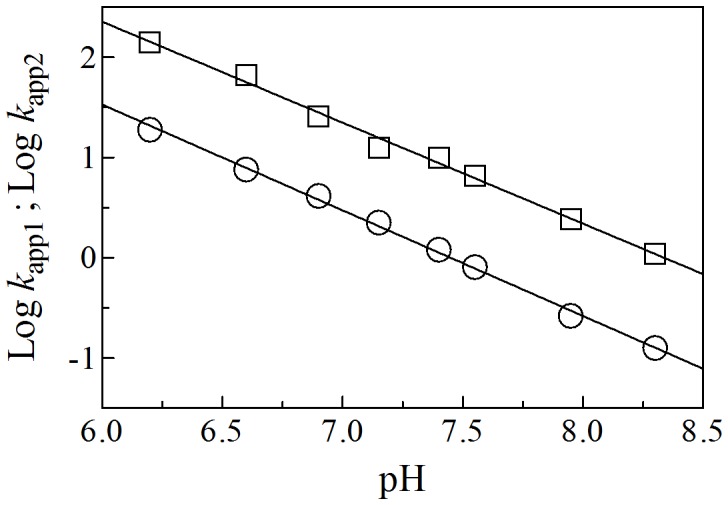
pH dependence of Log *k*_app1_ (M^–1^ s^–1^; squares) and Log *k*_app2_ (M^–1^ s^–1^; circles) for the Ma-Pgb*-Fe(II) reductase activity. The slope of the continuous lines is: –1.01±0.03 (squares) and –1.05±0.02 (circles). The standard deviation is smaller than the symbol. For details, see text.

As observed for several heme-proteins [34], [36]–[43], [45], [46], the NO_2_^–^-mediated conversion of Ma-Pgb*-Fe(II) to Ma-Pgb*-Fe(II)-NO requires one proton for the NO and OH^–^ formation (see Scheme 1). Accordingly, on increasing the proton concentration by one pH unit, the rates of the NO_2_^–^-mediated conversion of Ma-Pgb*-Fe(II) to Ma-Pgb*-Fe(II)-NO increase by one-order of magnitude (Fig. 5). The values of the slope of the linear fitting of Log *k*_app1_ and Log *k*_app2_ versus pH are –1.01±0.03 and –1.05±0.02 for the fast and the slow phase, respectively (Fig. 5). Unlike all the heme-proteins investigated [34], [36]–[43], [45], [46], values of *k*_app_ for the nitrite-reductase activity of human cytoglobin are essentially independent of pH between 8.5 and 7.0 (the average value being ∼0.14 M^–1^ s^–1^) and increase from 0.7 M^–1^ s^–1^ to 2.1 M^–1^ s^–1^ on lowering pH from 6.5 to 5.5 [44]. This behavior has been interpreted by taking into account the reversible pH-dependent penta-to-hexa-coordination transition of the heme-Fe(II) atom [44].

### The Ma-Pgb*-Fe(III)-mediated Peroxynitrite Isomerization

**Figure 6:**
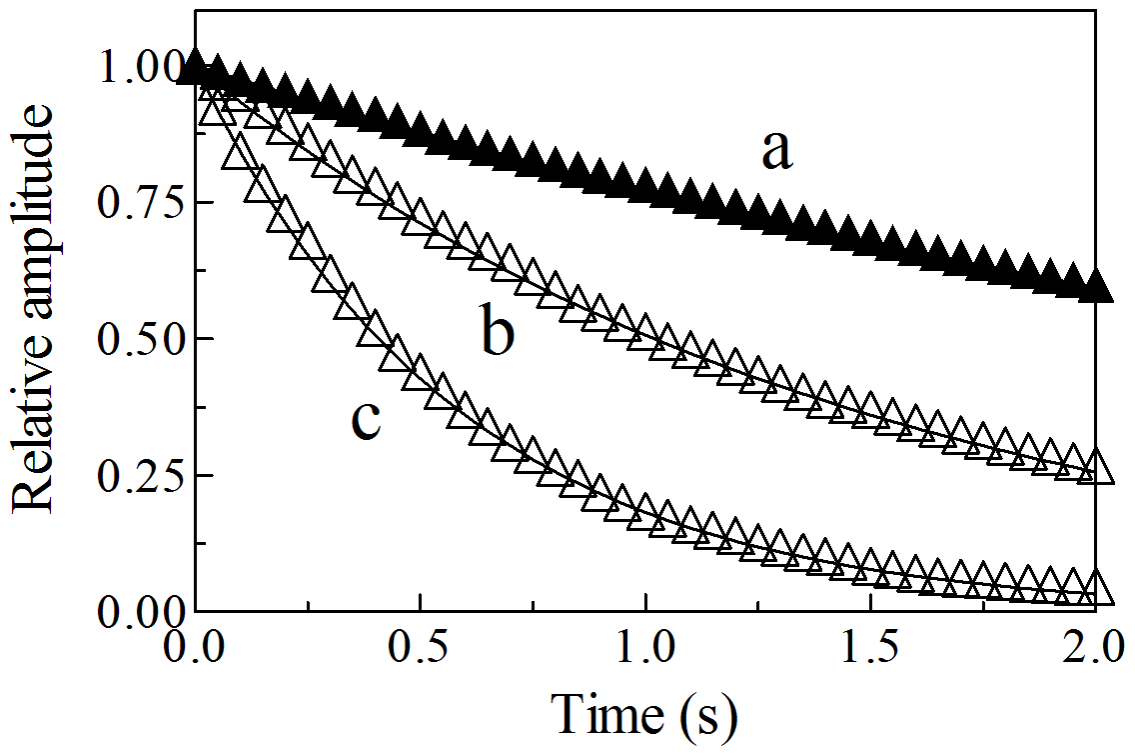
Normalized averaged time courses of the peroxynitrite isomerization by Ma-Pgb*-Fe(III) at 302 nm, in the absence and presence of azide, at pH 7.4 and 20°C. The Ma-Pgb*-Fe(III) concentration was 1.0×10^–5^ M (traces a and b) and 4.0×10^–5^ M (trace c). The azide concentration was 1.0×10^–1^ M (trace a). The peroxynitrite concentration was 2.5×10^–4^ M. Azide inhibits the peroxynitrite isomerization by Ma-Pgb*-Fe(III) (trace a). The time course analysis according to Eq. 4 allowed the determination of the following parameters: trace a, *h*_obs_ = 2.6×10^–1^ s^–1^; trace b, *h*_obs_ = 6.8×10^–1^ s^–1^; trace c, *h*_obs_ = 1.7 s^–1^. For details, see text.

Kinetics of peroxynitrite isomerization, both in the absence and presence of Ma-Pgb*-Fe(III) and Ma-Pgb*-Fe(III)-azide, was recorded by a single-wavelength rapid-mixing stopped-flow apparatus. Under all experimental conditions, a decrease of the absorbance at 302 nm was observed, as previously reported [21]–[32], [47], [48]. Kinetics of peroxynitrite isomerization was fitted to a single-exponential decay for more than 95% of its course at 302 nm and (Fig. 6). Of note, Ma-Pgb*-Fe(III) does not undergo any spectral change between 380 nm and 460 nm in the course of the Ma-Pgb*-Fe(III)-mediated peroxynitrite isomerization reaction. According to literature [21]–[25], [27]–[32], this suggests that no intermediate species (*e.g.*, Ma-Pgb*-Fe(III)-OONO; see Scheme 3) accumulate(s) in the course of peroxynitrite isomerization. Therefore, the formation of the transient Ma-Pgb*-Fe(III)-OONO species represents the rate-limiting step in catalysis, the conversion of Ma-Pgb*-Fe(III)-OONO to Ma-Pgb*-Fe(III) and NO_3_^−^ being faster by at least one order of magnitude.

**Figure 7:**
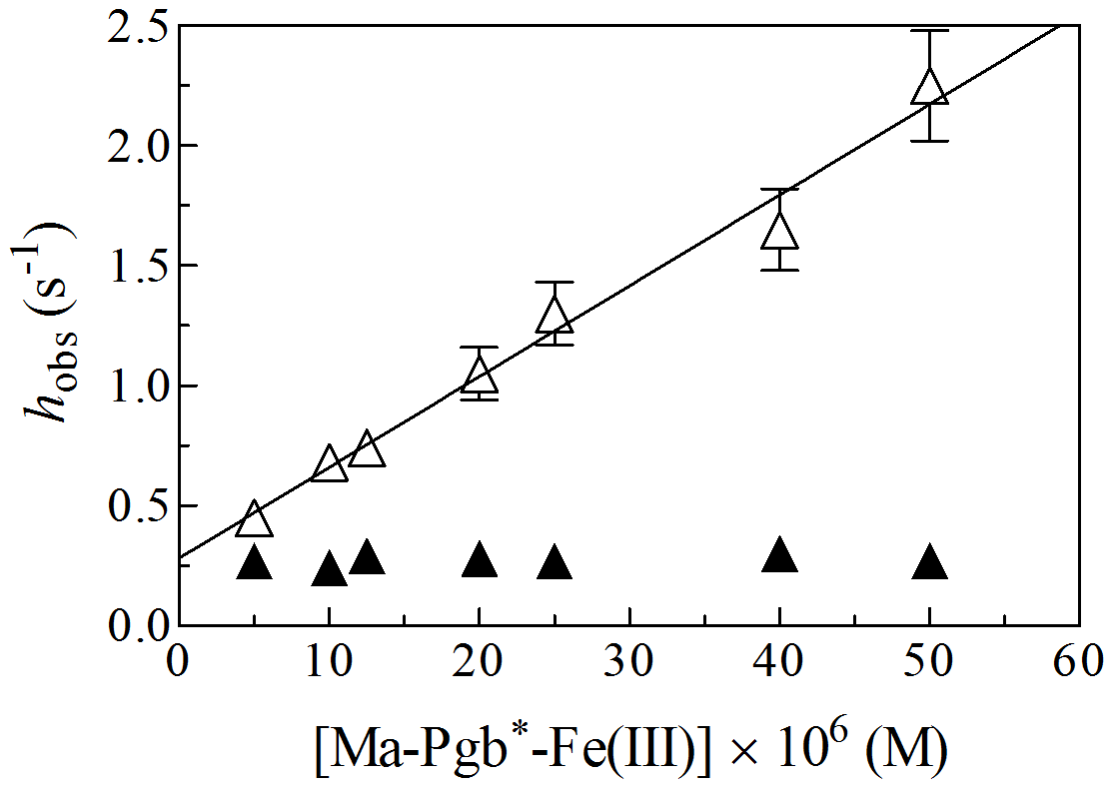
Dependence of *h* on the Ma-Pgb*-Fe(III) and Ma-Pgb*-Fe(III)-azide concentration (open and filled triangles, respectively) for the peroxynitrite isomerization, at pH 7.4 and 20°C. The continuous line was calculated according to Eq. 5 with *h*_app_ = 3.8×10^4^ M^–1^ s^−1^ and *k*_0_ = 2.8×10^–1^ s^−1^. The average value of *h*_0_ obtained in the presence of Ma-Pgb*-Fe(III)-azide (filled triangles) is 2.7×10^–1^ s^−1^. The peroxynitrite concentration was 2.5×10^–4^ M. The azide concentration was 1.0×10^–1^ M. Where not shown, the standard deviation is smaller than the symbol. For details, see text.

The observed rate constant for Ma-Pgb*-Fe(III)-mediated peroxynitrite isomerization (*i.e.*, *h*_obs_) increases linearly with the Ma-Pgb*-Fe(III) concentration (Fig. 7). The analysis of data reported in Fig. 7, according to Eq. 5, allowed the determination of values of the first-order rate constant for peroxynitrite isomerization in the absence of Ma-Pgb*-Fe(III) (*h*_0_ = 2.6×10^–1^ s^–1^ at pH 7.4), corresponding to the *y* intercept of the linear plot, and of the second-order rate constant for peroxynitrite isomerization by Ma-Pgb*-Fe(III) (*h*_on_ = 3.8×10^4^ M^−1^ s^−1^ at pH 7.4), corresponding to the slope of the linear plot. The value of *h*_0_ for peroxynitrite isomerization in the absence of Ma-Pgb*-Fe(III) ( = 2.8×10^–1^ s^−1^ at pH 7.4; Fig. 7) is in good agreement with those obtained in the presence of Ma-Pgb*-Fe(III)-azide ( = 2.7×10^–1^ s^−1^ at pH 7.4; Fig. 7) and in the absence of Ma-Pgb*-Fe(III) and Ma-Pgb*-Fe(III)-azide ( = 2.6×10^–1^ s^−1^ at pH 7.4; data not shown). Values of *h*_0_ here determined are in agreement with those reported in the literature [21]–[32], [47], [48]. Since Ma-Pgb*-Fe(III)-azide does not affect the peroxynitrite isomerization kinetics (Fig. 7), the acceleration of the peroxynitrite isomerization rate by Ma-Pgb*-Fe(III) must reflect the reaction of peroxynitrite with the heme-Fe(III) atom, as reported for ferric bacterial truncated hemoglobins [27], [28], [31], human hemoglobin [22], horse heart myoglobin [22], sperm whale myoglobin [24], human serum heme-albumin [28], [30], horse heart cytochrome *c* [29].

**Figure 8:**
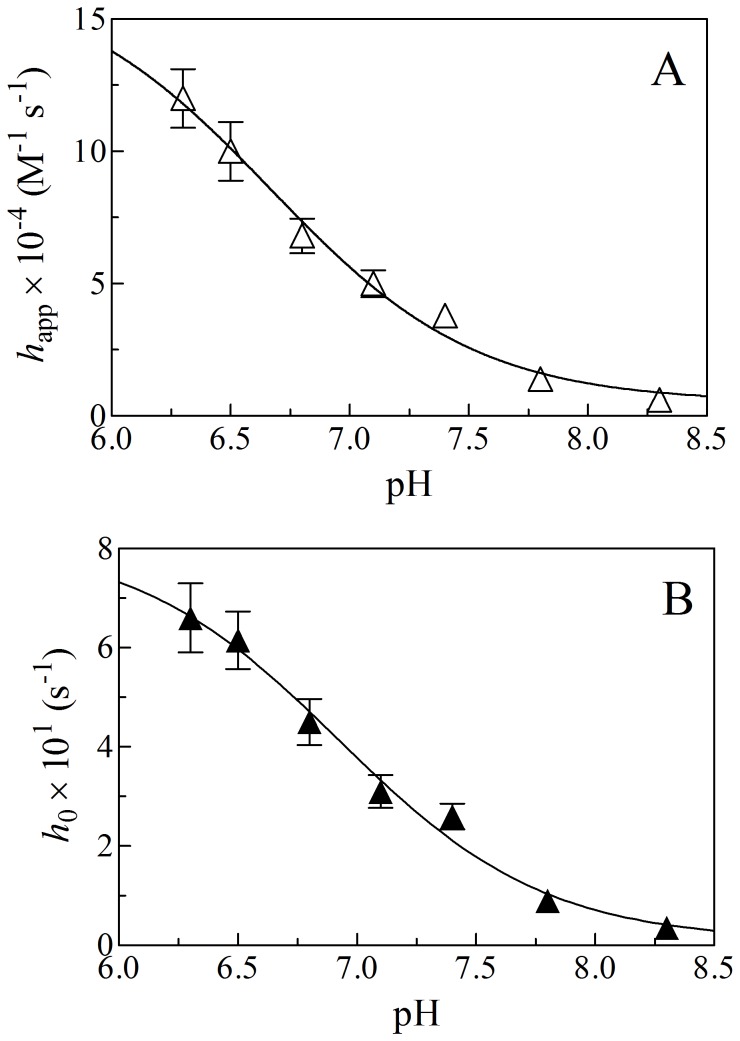
pH dependence of the second-order rate constant for the Ma-Pgb-Fe(III)-mediated peroxynitrite isomerization (*h*_app_; panel A) and of the first-order rate constant for the spontaneous isomerization of peroxynitrite to nitrate (*h*_0_; panel B), at 20°C. The continuous line in panel A was calculated according to Eq. 6 with p*K*_a_ = 6.7, *h*_app(prot)_ = 1.7×10^5^ M^−1^ s^−1^, and *h*_app(unprot)_ = 5.1×10^3^ M^−1^ s^−1^. The continuous line in panel B was calculated according to Eq. 6 with p*K*_a_ = 6.9, *h*_0(prot)_ = 8.2×10^−1^ s^−1^, and *h*_0(unprot)_ = 8.6×10^–3^ s^−1^. Where not shown, the standard deviation is smaller than the symbol. For details, see text.

To identify tentatively the species that preferentially react(s) with Ma-Pgb*-Fe(III), the effect of pH on kinetics of peroxynitrite isomerization in the absence and presence of Ma-Pgb*-Fe(III) (*i.e.*, on *h*_0_ and *h*_on_) was examined. As shown in Fig. 8, values of *h*_0_ and *h*_on_ increase on lowering pH from 8.3 to 6.2; the analysis of data, according to Eq. 6, allowed the estimation of p*K*_a_ values for the pH dependence of *h*_0_ and *h*_on_ values for peroxynitrite isomerization in the absence and presence of Ma-Pgb*-Fe(III). The pH dependence of *h*_0_ and *h*_on_ for peroxynitrite isomerization in the absence and presence of Ma-Pgb*-Fe(III) is similar, the p*K*_a_ values being 6.9 and 6.7, respectively (Fig. 8). The p*K*_a_ values for the pH dependence of *h*_0_ and *h*_on_ here determined are in excellent agreement with p*K*_a_ values reported in the literature [28], [48]. According to literature [22], [24], [28], the close similarity of the pH dependence of *h*_0_ for peroxynitrite isomerization in the absence of Ma-Pgb*-Fe(III) (p*K*_a_ = 6.9) (Fig. 8, panel A) and of *h*_app_ for peroxynitrite isomerization by Ma-Pgb*-Fe(III) (p*K*_a_ = 6.7) (Fig. 8, panel B) suggests that HOONO is the species that reacts preferentially with the heme-Fe(III) atom.

## Discussion

The observation, reported in this work, that Ma-Pgb* displays in the ferrous form (*i.e.*, Ma-Pgb*-Fe(II)) a nitrite-reductase activity and in the ferric form (*i.e.*, MaPgb*-Fe(III)) catalyzes peroxynitrite isomerization opens a new scenario on the physiological role played by this Pgb in the framework of *Methanosarcina acetivorans* metabolism. The efficiency of these two processes catalyzed by Ma-Pgb* indeed suggests that this heme-protein might play a role in the metabolism of reactive nitrogen and oxygen species facilitating, under reducing conditions, NO synthesis from NO_2_^–^ and, under oxidative conditions, peroxynitrite conversion to NO_3_^–^. In Table 1, the ligand-linked reactions of the reduced and oxidized forms of MaPgb* are summarized.

**Table 1:** Values of kinetic parameters for Ma-Pgb*-Fe reactivity.

Protein	Ligand	Kinetic parameters	
Ma-Pgb*-Fe(II) a	CO	*k*_on1_ = 1.9×10^7^ M^–1^ s^–1^	*k*_off1_ = 8.1×10^–2^ s^–1^
		*k*_on2_ = 3.1×10^6^ M^–1^ s^–1^	*k*_off2_ = 3.2×10^–2^ s^–1^
Ma-Pgb*-Fe(II) b	NO	*k*_on_ = 2.7×10^7^ M^–1^ s^–1^	
Ma-Pgb*-Fe(II) c	NO_2_^–^	*k*_app1_ = 9.6 M^–1^ s^–1^	
		*k*_app2_ = 1.2 M^–1^ s^–1^	
Ma-Pgb*-Fe(III) d	Azide	*k*_on_ ≥1×10^4^ M^–1^ s^–1^	*k*_off_ = 1.6×10^1^ s^–1^
Ma-Pgb*-Fe(III) e	Cyanide		*k*_off_ = 5.8×10^–5^ s^–1^
Ma-Pgb*-Fe(III) b	NO	*k*_on_ = 4.8×10^4^ M^–1^ s^–1^	*k*_off_ = 2.6 s^–1^
Ma-Pgb*-Fe(III) c	Peroxynitrite	*h*_app_ = 3.8×10^4^ M^–1^ s^–1^	*h*_0_ = 2.8×10^–1^ s^–1^

a pH 7.0 and 20°C. From [15].

b pH 7.2 and 22°C. From [16].

c pH 7.4 and 20°C. Present study.

d pH 7.0 and 20°C. From [18].

e pH 9.2 and 20°C. From [17].

Unlike all known globins, Ma-Pgb*-specific loops and the *N*-terminal extension completely bury the heme within the protein matrix (Fig. 1, panel A). Therefore, the access of ligands to the heme distal pocket is granted by the apolar tunnels reaching the heme distal site from locations at the B/G and B/E helix interfaces (Fig. 1, panel B) [11]. The presence of the two tunnels within the protein matrix may be partly responsible for the biphasic ligand binding behavior of Ma-Pgb*-Fe(II) towards NO_2_^–^ (present study) and CO [15], [18] (see Table 1). In contrast, azide binding to Ma-Pgb*-Fe(III) [18], cyanide dissociation from the Ma-Pgb*-Fe(III)-cyanide complex [17], Ma-Pgb*-Fe(III) nitrosylation [16], and Ma-Pgb*-Fe(III)-mediated peroxynitrite isomerization (present study) correspond to a monophasic process (see Table 1). Taken together, these data suggest the occurrence of the interplay between the oxidation state of the heme-Fe-atom and the modulation of ligand binding kinetics, which might be related to some conformational and/or dynamic differences between Ma-Pgb*-Fe(II) and Ma-Pgb*-Fe(III) and/or an oxidation state-linked different role of the apolar tunnels in modulating the ligand pathway. In this respect, TyrB10 and IleG11 residues, located in the heme distal site and lining the protein matrix tunnels 1 and 2, respectively, display a crucial role on the heme distal site structural organization and on the modulation of ligand binding to the heme-Fe-atom. In particular, the ligand accessibility to the heme distal site through tunnel 1 is modulated by the ligand-dependent reorganization of the TrpB9 and PheE11 side-chains, triggered by the TyrB10 and IleG11 residues. In this scenario, the PheE11 residue acts as the ligand sensor and controls the ligand accessibility to the heme distal pocket by modifying the conformation of the TrpB9 side chain [10], [14], [17]. Therefore, subtle different geometries of these residues in Ma-Pgb*-Fe(II) and Ma-Pgb*-Fe(III) could be reflected in a different regulation of ligand binding, as observed for kinetics of Ma-Pgb*-Fe(III) reactivity towards azide, cyanide, NO, and peroxynitrite (which is monophasic), and for kinetics of Ma-Pgb*-Fe(II) reactivity towards CO and NO_2_^−^ (which is biphasic) (see Table 1). In this respect, the only exception is represented by the monophasic very fast reaction of Ma-Pgb*-Fe(II) with NO (see Table 1) [16], which may either (*i*) reflect the loss of the first very fast step in the dead-time of the rapid-mixing stopped-flow apparatus, or else (*ii*) the existence of a ligand-linked conformational change(s) slower than NO binding and faster than CO binding, this resulting in a monophasic process for Ma-Pgb*-Fe(II) nitrosylation and of a biphasic process for Ma-Pgb*-Fe(II) carbonylation.

It should be underlined that NO_2_^–^ induces the conversion of Ma-Pgb*-Fe(II) to Ma-Pgb*-Fe(II)-NO whereas the Ma-Pgb*-Fe(II)-CO derivative does not react with NO_2_^–^, clearly indicating that the efficiency of the NO_2_^–^-mediated nitrosylation of Ma-Pgb*-Fe(II) requires an unliganded heme-Fe(II). From the comparative standpoint, kinetics of the NO_2_^–^-mediated conversion of wild type ferrous *Synechocystis* hemoglobin, *Arabidopsis thaliana* hemoglobin class 1 and 2, rice nonsymbiotic hemoglobin class 1, Ma-Pgb*-Fe(II), carp myoglobin-1 and -2, horse heart myoglobin, sperm whale myoglobin, mouse neuroglobin, human cytoglobin, human neuroglobin, human serum heme-albumin, and human hemoglobin to their ferrous nitrosylated derivatives (see Table 2) indeed reflects the different structural features of the heme site and/or of the structure-dependent energetic barriers along the ligand pathway toward the heme (see [37], [38], [41]–[46], [49] and present study).

**Table 2:** Values of the second-order rate constant for the nitrite-reductase activity of ferrous heme-proteins.

Heme-protein	*k*_app_ (M^–1^ s^–1^)
*Synechocystis* hemoglobin a	6.8×10^1^
*Arabidopsis thaliana* hemoglobin class 1 b	2.0×10^1^
*Arabidopsis thaliana* hemoglobin class 2 b	4.9
Rice nonsymbiotic hemoglobin class 1 a	3.3×10^1^
*Methanosarcina acetivorans* protoglobin* - fast c	9.6
– slow c	1.2
Carp myoglobin-1 d	5.3
Carp myoglobin-2 d	1.8
Horse heart myoglobin e	2.9
Sperm whale myoglobin f	6.0
Sperm whale myoglobin HisE7Ala mutant g	1.8
Sperm whale myoglobin HisE7Leu mutant g	<0.2
Mouse neuroglobin h	5.1
Human cytoglobin i	1.4×10^–1^
Human neuroglobin CysCD4-CysD5 j	1.2×10^–1^
Human neuroglobin CysCD4/CysD5 k	1.2×10^–1^
Human neuroglobin CysCD4Ala mutant l	5.8×10^–2^
Human neuroglobin CysD5Ala mutant l	6.0×10^–2^
Human neuroglobin HisE7Leu mutant l	2.6×10^2^
Human neuroglobin HisE7Gln mutant l	2.7×10^2^
Human hemoglobin - T state f	1.2×10^–1^
– R state f	6.0
Human serum heme-albumin m	1.3
Warfarin-bound human serum heme-albumin m	9.3×10^–2^
Horse heart cytochrome *c* n	7.0×10^–2^

a pH 7.0; unknown temperature [42].

b pH 7.4 and 25°C. From [45].

c CysE20Ser mutant. pH 7.4 and 20°C. Present study.

d pH 7.6 and 25°C. From [49].

e pH 7.4 and 25°C. From [43].

f pH 7.4 and 25°C. From [37].

g pH 7.4 and 25°C. From [43].

h pH 7.4 and 25°C. From [40].

i pH 7.0 and 25°C. From [44].

j pH 7.4 and 25°C. From [43]. In “Human neuroglobin CysCD4-CysD5”, the CysCD4 and CysD5 residues form an intramolecular disulphide bond.

k pH 7.4 and 25°C. From [43]. In “Human neuroglobin CysCD4/CysD5”, the CysCD4 and CysD5 residues do not form the intramolecular disulphide bond.

l pH 7.4 and 25°C. From [43].

m pH 7.4 and 20°C. From [46].

n pH 7.4 and 25°C. From [44].

The *k*_app_ values for the NO_2_^–^-mediated conversion of the ferrous deoxygenated HisE7Ala and HisE7Leu mutants of sperm whale myoglobin to their ferrous nitrosylated derivatives are significantly lower than that of the wild type protein, reflecting the different stabilization mode of the heme-Fe(II)-bound NO_2_^–^ (see Table 2) [43].

The low *k*_app_ values for the NO_2_^–^-mediated conversion of ferrous human cytoglobin to the ferrous nitrosylated derivative could reflect the hexa-coordination of the heme-Fe(II) atom (see Table 2) [44]. However, the NO_2_^–^-mediated conversion of ferrous human neuroglobin to the ferrous nitrosylated derivative reflects the reversible redox-linked hexa-to-penta-coordination transition of the heme-Fe(II) atom. Therefore, under oxidative conditions, the formation of the CysCD4-CysD5 bridge stabilizes the high-reactive penta-coordinated heme-Fe(II) atom facilitating the reaction. In contrast, under reductive conditions, the cleavage of the CysCD4-CysD5 bridge leads to the formation of the low-reactive hexa-coordinated heme-Fe(II) atom. Accordingly, the CysCD4Ala and CysD5Ala mutations, impairing the formation of the CysCD4-CysD5 bridge and stabilizing the hexa-coordinated heme-Fe(II) atom, slow down the NO_2_^–^-mediated conversion of ferrous human neuroglobin to its ferrous nitrosylated derivative. Also the HisE7Leu and HisE7Gln mutations, leading to a stable penta-coordinated heme-Fe(II) atom, facilitate the nitrite-reductase activity of ferrous human neuroglobin [43].

Although the heme-Fe atom of *Synechocystis* hemoglobin, *Arabidopsis thaliana* nonsymbiotic hemoglobins classes 1 and 2, and rice nonsymbiotic hemoglobin class 1 has been reported to be basically hexa-coordinated, *k*_app_ values for the NO_2_^–^-mediated conversion of the ferrous deoxygenated derivative to the ferrous nitrosylated species range between 4.9 M^–1^ s^–1^ and 6.8×10^1^ M^–1^ s^–1^ (see Table 2). This finding has been interpreted assuming that a substantial fraction of the ferrous derivative of these proteins may display a penta-coordinated heme geometry [42], [45]. In the case of Ma-Pgb*-Fe(II) nitrite reductase activity, the observed rate constants are *k*_app1_ = 9.6±0.2 M^–1^ s^–1^ and *k*_app2_ = 1.2±0.1 M^–1^ s^–1^; therefore, their values appear to fall in the average range observed for other heme-proteins, being somewhat slower that for *Synechocystis* hemoglobin, *Arabidopsis thaliana* nonsymbiotic hemoglobin class 1 and rice non symbiotic Hb class 1, but significantly faster than human cytoglobin and human neuroglobin (see Table 2). Such behavior suggests that the access of NO_2_^–^ to the heme, and heme reactivity, indeed meet some energetic barrier (due to the limited access through the apolar tunnels), but the heme itself should be mostly penta-coordinated.

Lastly, the NO_2_^–^-mediated conversion of ferrous human hemoglobin to its ferrous nitrosylated derivative is impaired allosterically by inositol hexakisphosphate binding to the central cavity of the tetramer, stabilizing the low reactive T-state (see Table 2) [37], [38]. Also the NO_2_^–^-mediated conversion of ferrous human serum heme-albumin to its ferrous nitrosylated derivative is inhibited allosterically by warfarin binding to the fatty acid binding site 2. In fact, warfarin binding to ferrous human serum heme-albumin induces the hexa-coordination of the heme-Fe atom which becomes unreactive (see Table 2) [46].

Peroxynitrite isomerization is facilitated by Ma-Pgb*-Fe(III), whereas the Ma-Pgb*-Fe(III)-azide derivative is non-reactive, clearly demonstrating that the efficiency of the isomerization process reflects the heme-Fe(III) reactivity; moreover, HOONO appears to be the species that preferentially reacts with Ma-Pgb*-Fe(III). Peroxynitrite isomerization by Ma-Pgb*-Fe(III), *Mycobacterium tuberculosis* truncated-hemoglobin N, *Pseudoalteromonas haloplanktis* TAC125 truncated-hemoglobin O, horse heart myoglobin, sperm whale myoglobin, human hemoglobin, human serum heme-albumin, cardiolipin-bound horse heart cytochrome *c* as well as cardiolipin-free and -bound carboxymethylated horse heart cytochrome *c* (see Table 3) represents a common feature of penta-coordinated heme-proteins (see [22], [24], [28], [29], [31], [32] and present study). In contrast, hexa-coordinated ferric horse heart cytochrome *c* and ferric human neuroglobin do not catalyze peroxynitrite isomerization [29], [50]. Notably, cardiolipin acts as an allosteric effector of horse heart cytochrome *c* inducing the cleavage of the sixth coordination bond of the heme-Fe atom (*i.e.*, Met80-Fe), thus stabilizing the penta-coordinated derivative [29].

**Table 3:** Values of the second-order rate constant for peroxynitrite isomerization by ferric heme-proteins.

Heme-protein	*h*_app_ (M^–1^ s^–1^)
*Methanosarcina acetivorans* protoglobin* a	3.8×10^4^
*Mycobacterium tuberculosis* truncated-hemoglobin N b	6.2×10^4^
*Pseudoalteromonas haloplanktis* TAC125 truncated-hemoglobin O c	2.9×10^4^
Horse heart myoglobin d	2.9×10^4^
Sperm whale myoglobin e	1.6×10^4^
Sperm whale myoglobin HisE7Ala mutant e	5.8×10^6^
Sperm whale myoglobin HisE7Asp mutant e	4.8×10^6^
Sperm whale myoglobin HisE7Leu mutant e	5.7×10^4^
Sperm whale myoglobin PheCD1Trp/HisE7Leu mutant e	5.2×10^4^
Sperm whale myoglobin HisE7Tyr/HisF8Gly mutant e	9.0×10^3^
Human hemoglobin f	1.2×10^4^
Human serum heme-albumin g	4.1×10^5^
Cardiolipin-bound horse heart cytochrome *c* h	3.2×10^5^
Carboxymethylated horse heart cytochrome *c* h	6.8×10^4^
Cardiolipin-bound carboxymethylated horse heart cytochrome *c* h	5.3×10^5^

a pH 7.4 and 20°C. Present study.

b pH 7.0 and 20°C. From [32].

c pH 7.0 and 20°C. From [31].

d pH 7.0 and 20°C. From [22].

e pH 7.5 and 20°C. From [24].

f pH 7.5 and 20°C. From [22].

g pH 7.2 and 22°C. From [28].

h pH 7.0 and 20°C. Cardiolipin was 1.6×10^–4^ M. From [29].

The value of *h_on_* for peroxynitrite isomerization by MaPgb*-Fe(III) (see Table 3) falls in the same range as observed for other heme-proteins from different sources (such as *Mycobacterium tuberculosis* truncated-hemoglobin N and mammalian myoglobins and hemoglobins; see Table 3), clearly indicating that the binding process is closely similar, and confirming that MaPgb*-Fe(III) is essentially penta-coordinated [10], [11]. In this respect, the analysis of kinetics for peroxynitrite isomerization by ferric sperm whale myoglobin mutants (see Table 3) suggests that the heme-Fe(III) reactivity towards peroxynitrite is regulated either by steric factors modulating the ligand accessibility to the metal center (in the absence of a hydrogen-bonding residue, *i.e.* His E7) or by the Lewis acidity of the heme-Fe(III) atom [24]. Thus, the two most active mutants of ferric sperm whale myoglobin are HisE7Ala and HisE7Asp, which bind a water molecule as the sixth ligand of the heme-Fe(III) atom that however is not stabilized by hydrogen bonding to any heme distal residue, in contrast to what occurs in the ferric wild-type heme-protein. Although the HisE7Leu mutant of sperm whale myoglobin does not bind a water molecule at the sixth coordination position of the heme-Fe(III) atom, it displays a reactivity lower than that of the His64Ala and HisE7Asp mutants. This may reflect the steric hindrance exerted by the Leu residue, limiting the peroxynitrite accessibility to the heme-Fe(III) center. Moreover, the substitution of PheCD1 with the larger Trp residue, in the heme distal pocket, slightly reduces the reactivity of the heme-Fe(III) atom toward peroxynitrite. The low reactivity of the HisE7Tyr/HisF8Gly mutant has been ascribed to either the low accessibility of peroxynitrite to the proximal side of the heme, or the reduced Lewis acidity of the heme-Fe(III) atom as a consequence of TyrE7 residue binding to the heme-Fe atom. The role of steric factors modulating peroxynitrite accessibility to the metal center appears to be highlighted by the correlation between the values of second-order rate constant for azide binding to ferric HisE7Ala and HisE7Leu mutants and wild-type sperm whale myoglobin (2×10^6^ M^–1^ s^–1^, 3.4×10^4^ M^–1^ s^–1^, and 2.9×10^3^ M^–1^ s^–1^, respectively), which are strongly influenced by the size of the heme distal residues [51], and the values of *h*_on_ for the heme-based conversion of peroxynitrite to NO_3_^–^ (see Table 3) [24]. It is worth noting that also azide binding to Ma-Pgb*-Fe(III) displays a behavior closely similar to what has been observed for other heme-proteins [18], indeed suggesting that in the ferric form steric factors posed by the two apolar tunnels do not dramatically alter the energetics of the ligand binding pathway.

## Conclusions

Ma-Pgb shows a selectivity ratio for O_2_/CO binding that favors O_2_ ligation [11] and displays anti-cooperativity in CO binding [15]. This very unusual behavior could be related to the fact that *Methanosarcina acetivorans* takes advantage of acetate, methanol, CO_2_ and CO as carbon sources for methanogenesis; methane production occurs simultaneously with the formation of a proton gradient that is essential for energy harvesting [9], [52], [53]. Therefore, the capability to convert CO to methane suggests that CO is the actual ligand of Ma-Pgb *in vivo*, this being in agreement with the hypothesis of the very ancient origin for this metabolic pathway(s) [9], [54].

However, an additional Ma-Pgb role, which has been already postulated for other bacterial heme-proteins, is that of detoxifier to preserve the environment free of oxygen and reactive nitrogen and oxygen species. This hypothesis, which is obviously not in contrast with the metabolic role of Ma-Pgb, is supported by the present data. We show that Ma-Pgb* is able to play such scavenging role both in the reduced form, whereby under reducing environmental conditions Ma-Pgb*-Fe(II) may behave as a nitrite-reductase, and in the oxidized form, Ma-Pgb*-Fe(III) being able to catalyze the isomerization of peroxynitrite to nitrate in an oxidizing atmosphere. Such multiple roles may indeed reflect the adaptation of this ancient protein to different environmental conditions met during evolution.

## Supporting Information

Figure S1 Time courses of the NO_2_^–^-mediated conversion of Ma-Pgb*-Fe(II) to Ma-Pgb*-Fe(II)-NO, at pH 7.4 and 20°C. The observation wavelength was 445 nm (trace a), 435 nm (trace b), 425 nm (trace c), 420 nm (trace d), and 415 nm (trace e). Trace a was analyzed according to Eq. 1a with [Ma-Pgb*-Fe(II)]_i1_ = 14.24 mM^–1^ cm^–1^ and [Ma-Pgb*-Fe(II)]_i2_ = 10.75 mM^–1^ cm^–1^. Trace b was analyzed according to Eq. 1a with [Ma-Pgb*-Fe(II)]_i1_ = 31.94 mM^–1^ cm^–1^ and [Ma-Pgb*-Fe(II)]_i2_ = 24.08 mM^–1^ cm^–1^. Trace c was analyzed according to Eq. 1a with [Ma-Pgb*-Fe(II)]_i1_ = 3.41 mM^–1^ cm^–1^ and [Ma-Pgb*-Fe(II)]_i2_ = 2.57 mM^–1^ cm^–1^. Trace d was analyzed according to Eq. 1b with [Ma-Pgb*-Fe(II)]_i1_ = 16.55 mM^–1^ cm^–1^ and [Ma-Pgb*-Fe(II)]_i2_ = 12.46 mM^–1^ cm^–1^. Trace e was analyzed according to Eq. 1b with [Ma-Pgb*-Fe(II)]_i1_ = 33.65 mM^–1^ cm^–1^ and [Ma-Pgb*-Fe(II)]_i2_ = 25.36 mM^–1^ cm^–1^. At all wavelengths, values of *k*_obs1_ and *k*_obs2_ were 7.9×10^–2^ s^–1^ and 9.0×10^–3^ s^–1^. The NO_2_^–^ concentration was 8.0×10^–3^ M.
(TIF)

Figure S2 Difference absorbance spectrum of Ma-Pgb*-Fe(II)-CO ***minus*** Ma-Pgb*-Fe(II)-NO, at pH 7.4 and 20°C.
(TIF)
