# Supplementary figures and images for: Nitrite-Reductase and Peroxynitrite Isomerization Activities of Methanosarcina acetivorans Protoglobin
Source: PLoS One. 2014 May 14;9(5):e95391. doi: 10.1371/journal.pone.0095391 (PMC4020757; doi:10.1371/journal.pone.0095391)

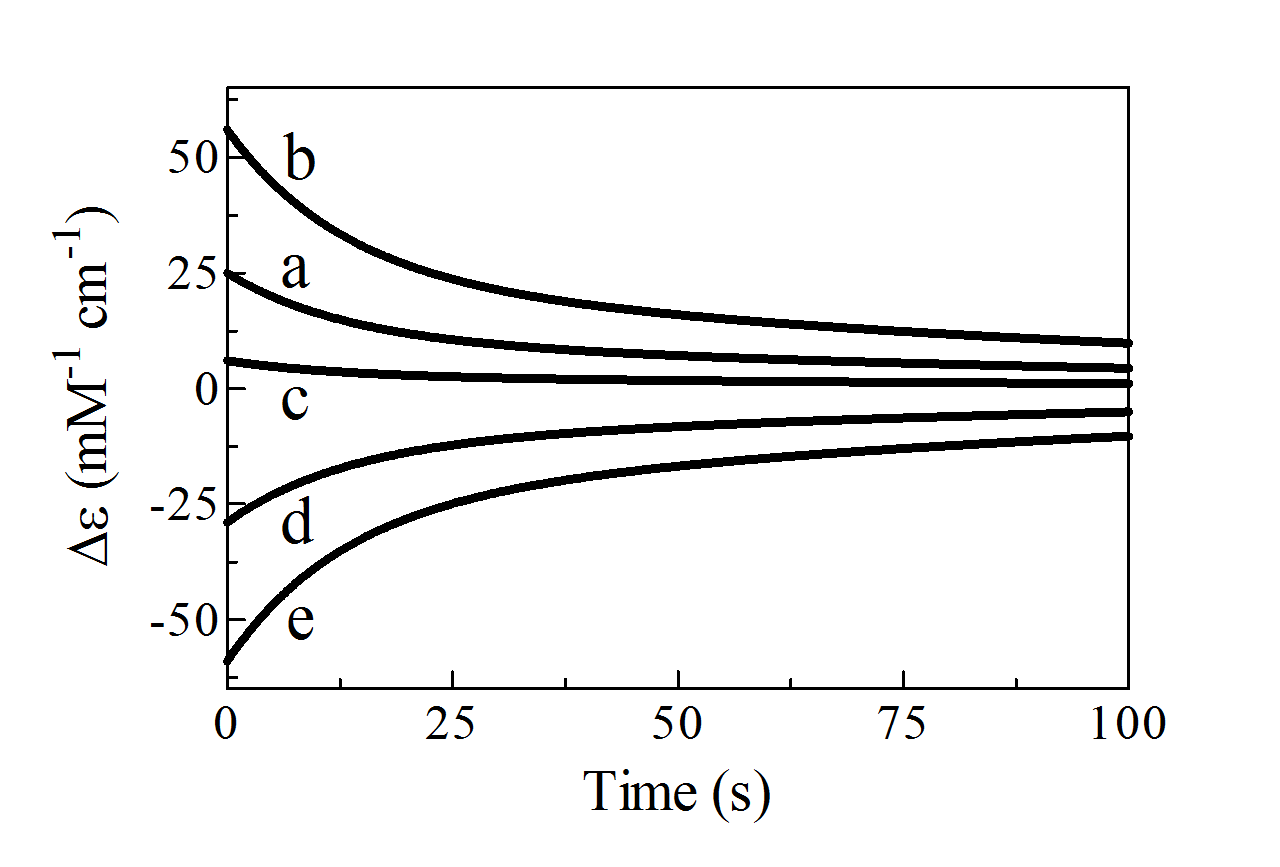

Supplement: Figure S1 — Time courses of the NO2–-mediated conversion of Ma-Pgb*-Fe(II) to Ma-Pgb*-Fe(II)-NO, at pH 7.4 and 20°C. The observation wavelength was 445 nm (trace a), 435 nm (trace b), 425 nm (trace c), 420 nm (trace d), and 415 nm (trace e). Trace a was analyzed according to Eq. 1a with [Ma-Pgb*-Fe(II)]i1 = 14.24 mM–1 cm–1 and [Ma-Pgb*-Fe(II)]i2 = 10.75 mM–1 cm–1. Trace b was analyzed according to Eq. 1a with [Ma-Pgb*-Fe(II)]i1 = 31.94 mM–1 cm–1 and [Ma-Pgb*-Fe(II)]i2 = 24.08 mM–1 cm–1. Trace c was analyzed according to Eq. 1a with [Ma-Pgb*-Fe(II)]i1 = 3.41 mM–1 cm–1 and [Ma-Pgb*-Fe(II)]i2 = 2.57 mM–1 cm–1. Trace d was analyzed according to Eq. 1b with [Ma-Pgb*-Fe(II)]i1 = 16.55 mM–1 cm–1 and [Ma-Pgb*-Fe(II)]i2 = 12.46 mM–1 cm–1. Trace e was analyzed according to Eq. 1b with [Ma-Pgb*-Fe(II)]i1 = 33.65 mM–1 cm–1 and [Ma-Pgb*-Fe(II)]i2 = 25.36 mM–1 cm–1. At all wavelengths, values of k obs1 and k obs2 were 7.9×10–2 s–1 and 9.0×10–3 s–1. The NO2 – concentration was 8.0×10^–3^ M. (TIF) [file pone.0095391.s001.tif]

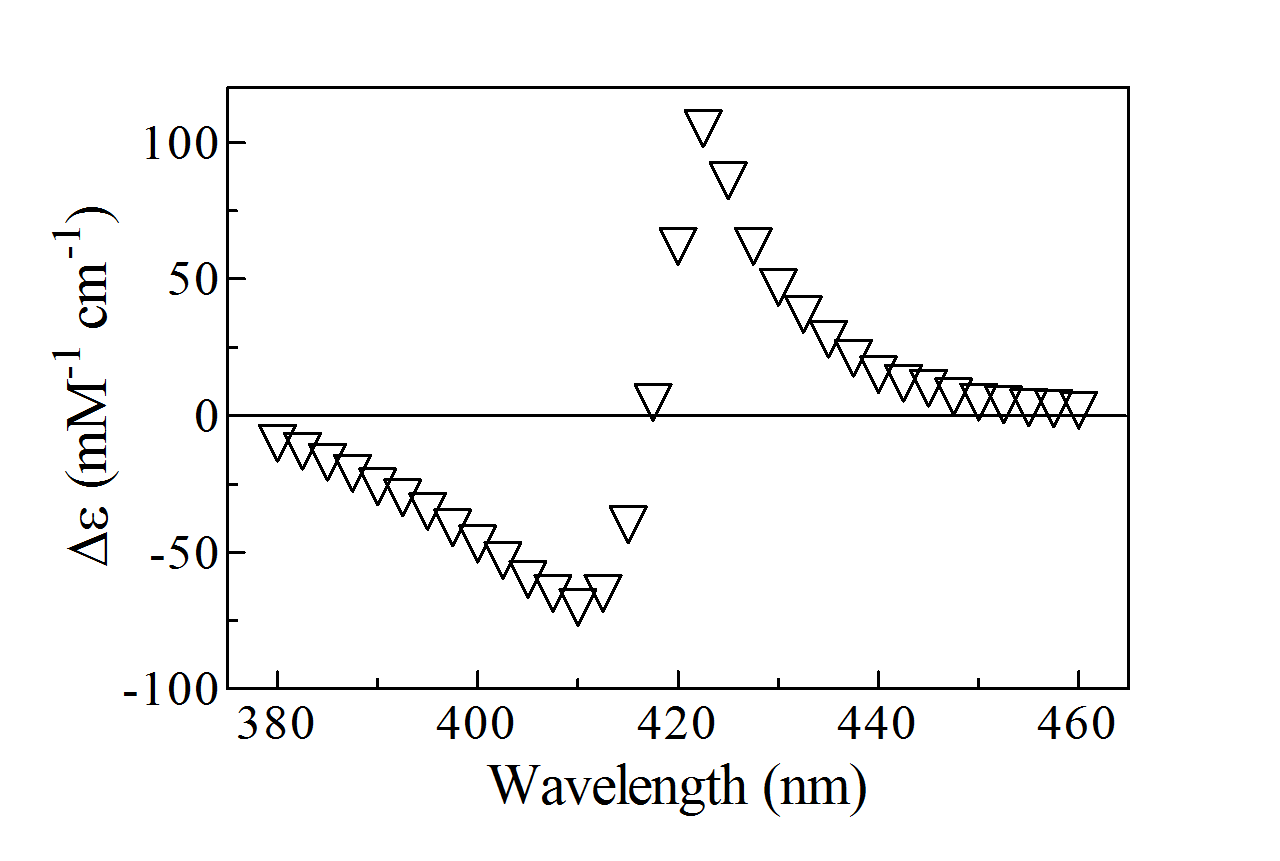

Supplement: Figure S2 — Difference absorbance spectrum of Ma-Pgb*-Fe(II)-CO minus Ma-Pgb*-Fe(II)-NO, at pH 7.4 and 20°C. (TIF) [file pone.0095391.s002.tif]
